# Fecal Metabolites As Non-Invasive Biomarkers of Gut Diseases

**DOI:** 10.32607/actanaturae.10954

**Published:** 2020

**Authors:** E. S. Zhgun, E. N. Ilina

**Affiliations:** Federal Research and Clinical Center of Physical-chemical Medicine of Federal Medical Biological Agency, Moscow, 119435 Russia

**Keywords:** fecal metabolites, non-invasive biomarkers, inflammatory bowel diseases, metabolomic analysis

## Abstract

Recent studies have shown the importance of the human intestinal microbiome in maintaining a healthy gastrointestinal tract, as well as in the development of pathological processes. The intestinal microbiome manifests itself primarily as fecal metabolites. In the past decade, there has been growing interest in studying its composition, which for the most part had to do with the possibility of using the metabolomic analysis in clinical diagnosis. In contrast to the comprehensive description of blood serum, urine, saliva, and cerebrospinal fluid metabolites, data on fecal metabolites is sparse. Despite the instrumental and methodological achievements in the metabolomic analysis in general, the analysis of fecal metabolome remains less well developed, mainly because of the inhomogeneity of its composition and the lack of standardized methods for collecting, processing, and analyzing fecal samples. This review summarizes data on methods for studying and describing various groups of fecal metabolites. It also assesses their potential as tools in the diagnosis of gastrointestinal diseases.

## INTRODUCTION

Despite the rapid development of analysis tools and the accumulation of data on human metabolites in general, feces remain poorly studied. Fecal composition is very complex and heterogeneous. The bulk of the solid fraction (84 to 93%) consists of organic material, 25–54% of which is bacterial biomass represented by both living and dead bacteria [1]. For this reason, most studies on the composition of human feces seek to identify their bacterial component by high-throughput sequencing. However, feces also contain cell masses, large and small molecules produced as a result of food consumption, digestion, as well as subsequent absorption by both the gastrointestinal tract (GIT) and intestinal bacteria. The macromolecules include macrofibers, proteins, DNA, polysaccharides, etc. Sugars, organic acids, amino acids, nucleotides, vitamins, and volatile organic compounds (VOCs) belong to the class of small molecules forming the intestinal metabolome. The use of an integrative Fecal Metabolites As Non-Invasive Biomarkers of Gut Diseases E. S. Zhgun*, E. N. Ilina Federal Research and Clinical Center of Physical-chemical Medicine of Federal Medical Biological Agency, Moscow, 119435 Russia *E-mail: Al.androva@gmail.com Received December 26, 2019; in final form, March 04, 2020 DOI: 10.32607/actanaturae.10954 Copyright ® 2020 National Research University Higher School of Economics. This is an open access article distributed under the Creative Commons Attribution License,which permits unrestricted use, distribution, and reproduction in any medium, provided the original work is properly cited. approach including a comprehensive analysis of fecal metabolites can significantly expand information on its composition [2, 3]. Determination of the metabolic profile is increasingly used to search for new biological markers of various pathological conditions and offer new hypotheses regarding their origin.

**Fig. 1:**
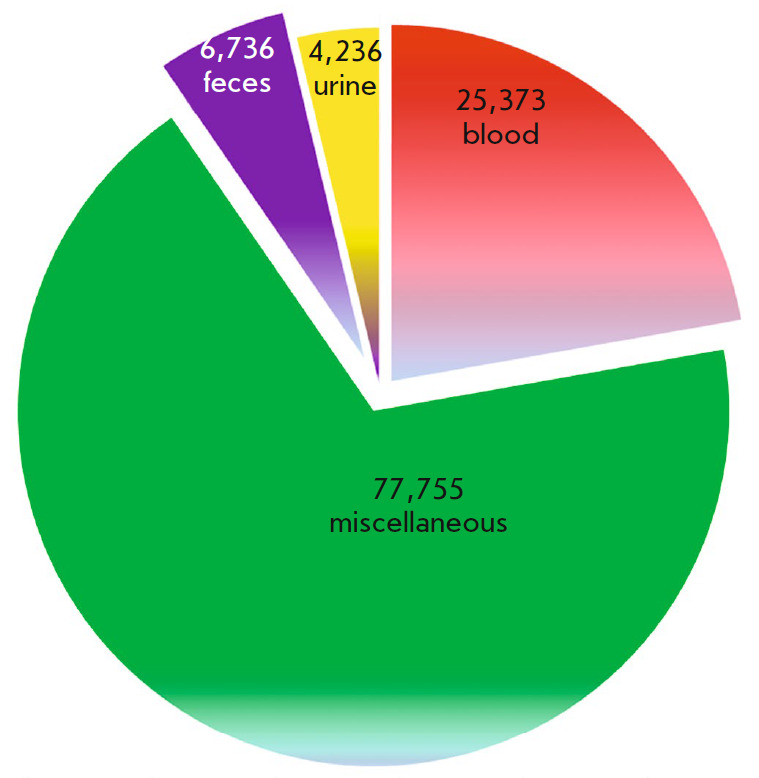
Comparison of a number of known human metabolites in various biological substances (according to the Human Metabolome Database (HMDB), http://www.hmdb.ca/)

Metabolites of serum [4], urine [5], cerebrospinal fluid [6], and saliva [7] have been the most fully described and characterized, among others. They have allowed for the creation of public reference databases for these metabolites [8]. To date, the Human Metabolome Database (http://www.hmdb.ca/) [8] contains information on more than 100,000 compounds, > 25,000 of which are blood metabolites, while urine and fecal components comprise over 4,000 and around 7,000 of the compounds, respectively (*Fig. 1*). The concentration of almost each of these 100,000 metabolites can change under various pathological conditions. However, only reproducible changes in the metabolite production can be used as a disease marker. Modern instrumental methods of analysis allow one to determine both individual metabolites and metabolic profiles. Hypo- and overproduction of a specific metabolite do not always clearly correlate with disease severity, which means that the metabolite cannot be considered a marker. However, inclusion of a substance in the panel of metabolites typical of a specific disease adds more diagnostic value to it [9, 10].

To date, 6,736 fecal metabolites have been described, comprising 5.9% of the total number of characterized metabolites. It is extremely important to study their potential as non-invasive diagnostic markers, since they can specifically identify intestinal processes and be associated with certain diseases of the large intestine, colon, and rectum [11, 12].

Human feces have been studied for thousands of years. Doctors in ancient China, Egypt, ancient Greece and Rome evaluated intestinal and hepatic function by the stool color and odor and then adjusted a patient’s diet [13]. Today, the advances in medicine allow one to use various quantitative fecal tests. They include the fecal pH test for assessing the content of fatty acids, malabsorption of carbohydrates, and for detecting lactose intolerance; detection of intestinal bacterial infections or the toxins produced by them (*Clostridium difficile *and *Helicobacter pylori*) by immunological methods and molecular genotyping [14, 15]; and the use of certain fecal proteins, especially calprotectin, for the diagnosis and monitoring of inflammatory bowel diseases [16]. Fecal occult blood testing is used to quickly detect gastrointestinal bleeding and colon cancer in its early stages [17]. In addition, neoplasm-specific changes have been characterized in DNA recovered from stool. They could act as potential markers of colorectal cancer [18]. A portable gas-sensing electronic nose system was created for detecting a set of fecal VOCs and diagnosing a number of pathological conditions, including cancer [19].

**Fig. 2:**
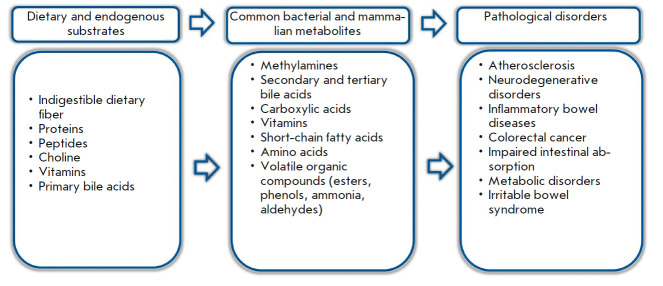
Human intestinal metabolites and their relationship with the host organism

Although interest in the study of fecal metabolites keeps growing, standardized methods for collecting, processing, and analyzing fecal samples are still lacking. Feces are quite difficult to study, since they are heterogeneous in composition, multicomponent, and rich in macromolecules and particles of undigested food, which can complicate their analysis using instrumental methods. The composition of fecal metabolites varies greatly depending on the type of food consumed and is a product of co-metabolism by both host and intestinal microorganisms (*Fig. 2*). Unlike urine [5], serum [4], cerebrospinal fluid [6], and saliva [7], fecal metabolism has never been examined systematically. However, knowledge extracted from the analysis of metabolites from various biomaterials significantly facilitates the optimization of a fecal analysis and provides important quantitative control means of comparison and distinguishing between disease and health.

**Fig. 3:**
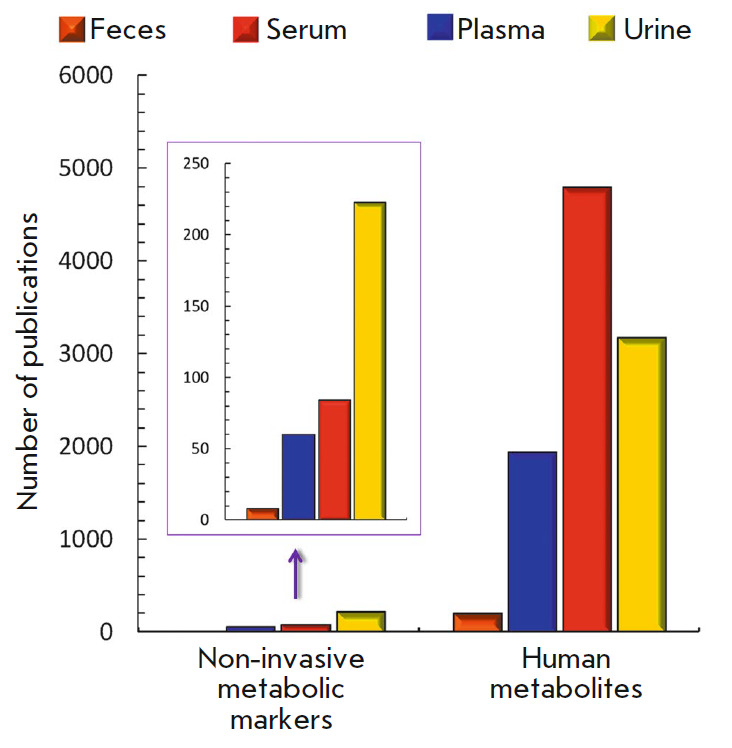
Comparison of the number of publications on non-invasive metabolic markers with the number of publications on human metabolites according to https://www.ncbi.nlm.nih.gov/pubmed for 2019

*Figure 3* presents data on the number of studies of the metabolites and non-invasive metabolic biomarkers in the most essential human biological substances, such as feces, serum, plasma, and urine for the period of 2010–2018. The figure shows that plasma and urine are the most studied biomaterials: they are mentioned in 4,793 and 3,172 publications, respectively, while feces are much less studied (only 198 articles). It is interesting to note that, although almost 40% more metabolites have been identified in feces than urine, there exist 15 times fewer papers on the study of stool metabolites. This imbalance is also noted in other biological substances (serum, plasma). The number of articles on non-invasive metabolic markers correlates with the total number of publications (*Fig. 3*). Thus, metabolic markers were studied in 7% of the papers on human urine metabolites, 1.8% of the articles on plasma metabolites, and in 3% and 4% of publications regarding serum and feces, respectively. One can assume that the small number of markers detected in feces is only due to the low interest the world scientific community has in its study.

**Instrumental analysis of fecal samples**

The study of fecal metabolites is a difficult analytical undertaking, since molecules within the intestinal content are of both endogenous (human- and microorganism recovered) and exogenous origin [20]. The latter compounds include ingested, absorbed or inhaled materials (food components, gases and smoke, personal hygiene products, preservatives, and other materials) humans are exposed to on a daily basis [20].

The main methods used to study fecal metabolites are chromatography, mass spectrometry, and NMR. A method’s sensitivity and metabolite coverage vary significantly depending on the type of analytical tool, since different platforms vary in their sensitivity to different classes of metabolites. For instance, gas chromatography (GC–S) is most effective for detecting volatile and organic compounds, while NMR and liquid chromatography (LC–S) are more suitable to very polar and hydrophobic substances, respectively. Therefore, it is ideal to use more than one platform in order to achieve maximum metabolite coverage. A combination of two or more analytical platforms has been used in around 15% of the more than 100 studies published to date.

**Nuclear magnetic resonance**

NMR, and 1H NMR in particular, is widely used to detect metabolites in biological samples. The method has several advantages over LC–MS and GC–MS chromatography, such as a very high reproducibility, reliable compound identification/classification, minimal sample preparation without chemical derivatization, and the possibility of detecting non-ionic compounds (such as sugars and alcohols) without disrupting their structure. The disadvantages include lower sensitivity compared to mass spectrometric identification (up to 1,000 times at the molar level), which significantly narrows the application of this method [21-23].

NMR is most often used to detect amino acids and their derivatives, carboxylic acids, including short- and medium-chain fatty acids and their derivatives, as well as sugars and bile acids.

**Methods of mass spectrometry analysis**

To date, mass spectrometry analysis is the most viable alternative to NMR. MS analysis can be either direct or coupled with preliminary separation by GC–MS, LC–MS chromatography or capillary electrophoresis (the latter is currently extremely rare).

Mass spectrometry analysis can be targeted and untargeted. The targeted approach is aimed at identifying specific classes of metabolites (e.g. amino acids, fatty acids, lipids, carbohydrates, and bile acids), while the second is used to collect general information on the metabolic diversity of the sample; the so-called metabolic profile [20, 23].

Each approach has its own unique advantages. Targeted mass spectrometry is generally more sensitive and allows one to obtain more quantitative results; however, it is limited to the identification of certain classes of molecules. Using more than 100 fecal samples, the American Gut project demonstrated that a targeted analysis incorrectly identifies up to 30% of the primary data and can lead to their misinterpretation [24].

On the contrary, the untargeted analysis allows one to identify a wide range of molecules and, therefore, potentially discover new, previously unknown, molecules. However, identifying the obtained spectra remains one of the challenges [25]. This issue can be partially resolved by finding matches in the existing mass spectra and compound databases, such as HMDB [8], METLIN [26] or ChemSpider [27], as well as in the metabolic pathway databases KEGG [28] and MetaCyc [29]. On average, only 2% of untargeted LC–MS data are annotated [28-31].

A targeted analysis also provides for better correlation with the microbiome data, thus allowing one to determine the relationship between the microorganisms and the metabolites they produce or utilize [20].

Software that allows to process targeted and untargeted analysis data has been developed; it enables a more comprehensive and unbiased characterization of a sample’s metabolic composition and its functional relationship to the microbiome [20].

GC–MS chromatography is the most commonly used analytical method for studying fecal metabolites. GC–MS gained popularity thanks to the wide range of metabolites it allows to detect, its high sensitivity, and the relative simplicity of its compound identification. GC–MS is used to analyze volatile and non-volatile organic compounds (with preliminary chemical derivatization of the compounds to improve their volatility).

Liquid chromatography, coupled with mass spectrometry (LC–MS), is less commonly used than GC– MS is in fecal metabolomics, which has to do with the lower chromatographic efficiency of LC–MS compared to GC–MS in terms of peak shape and resolution.

Chromatographic approaches remain the most relevant methods of analysis for some groups of fecal metabolites: in particular, short-chain fatty acids (SCFAs). Gas chromatography (GC), which has been in use in clinical diagnosis since 1952, remains the gold standard [32]. The principle behind GC is based on the use of a carrier gas as the mobile phase in which the compounds are separated by differential interaction with the column’s stationary phase [33].

Pre-treatment of feces [34, 35], including filtration, centrifugation, steam/vacuum distillation or simple dilution of the sample [33, 36, 37], play a crucial role in the qualitative and quantitative detection of SCFAs. Derivatization of SCFAs, which is necessary in order to improve compound volatility, is achieved by deprotonation; i.e., acidification with hydrochloric [38], phosphoric [39], formic [40], sulfuric [41], or oxalic [42] acid.

In addition to extraction with various solvents, which allows to separate two immiscible layers, solidphase microextraction (SPME) is also an effective approach; it is quickly becoming a faster, more selective and sensitive technique thanks to fewer impurities [43].

High-performance liquid chromatography (HPLC) is a good alternative to GC. Reverse-phase HPLC, in which the stationary solid phase (column) is hydrophobic and the mobile liquid phase is hydrophilic, is the most commonly used. Its main advantage compared to GC resides in the absence of high temperatures. As in the case of GC, the method requires an optimization of sample preparation and experimental conditions for a successful analysis [44, 45].

**The human fecal metabolome database HFMDB**

**Fig. 4:**
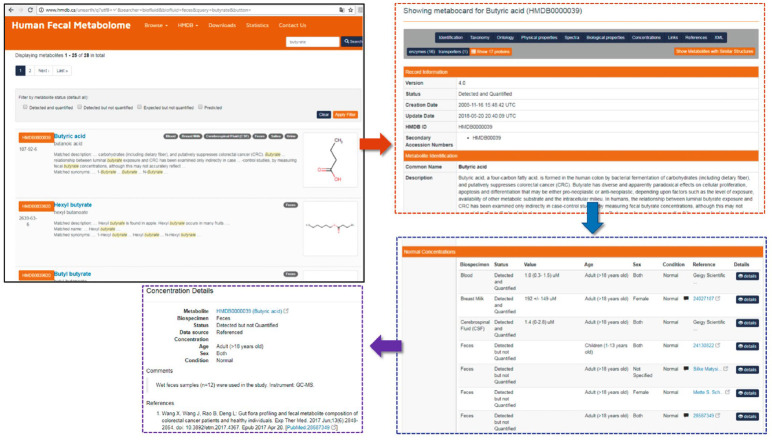
Screenshot of the Human Fecal Metabolome Database (HFMDB) (http://www.fecalmetabolome.ca)

A list of 1,890 compounds covering most known metabolite classes has been created based on the results of the approximately 100 studies of fecal metabolites published to date. The total number of metabolites (including isomers) comprising an open-source database (http://www.fecalmetabolome.ca) is 6,738 (*Fig. 4*) [46]. Each fecal metabolite has its individual number and is listed in the Human metabolome database (HMDB) [8]. The HMDB contains a detailed description of each metabolite, including its structure, chemical taxonomy information, known synonyms, physicochemical properties, reference NMR, GC–MS, and LC–MS spectra, as well as the association with diseases and the possible metabolic pathways it is involved in. The database also provides metabolite concentrations in feces and other biological samples (if there are any) with the corresponding normal range values.

## METABOLITES IN THE HUMAN GUT

According to the HMDB, intestinal bacterial products account for a large proportion (up to 92%) of the fecal metabolome, while they comprise only 3% of the urine metabolome [5]. Intestinal microbial metabolites include short- and medium-chain fatty acids, amino acids and their derivatives, alcohols, aldehydes, phenols and polyphenol derivatives, as well as indoles and sulfides [47].

The most common human fecal metabolites (normalized to stool weight) are SCFAs: acetic (36 ± 17 μmol/g), propionic (11 ± 5 μmol/g), and butyric (6 ± 3 μmol/g) acids and their isoforms (according to the targeted GC–S data [48]), while lipids are the least common. Phosphatidylcholines are found at a level below 0.02 ± 0.01 nmol/g of wet feces (LC–S/MS data [3]). Acylcarnitines (LC–S/MS data [3]), secondary bile acids, tauroursodeoxycholic acid (0.3 ± 0.37 nmol/g of wet feces), and lithocholic acid taurine conjugate (0.51 ± 0.4 nmol/g of wet feces) are also present in small concentrations (LC–MS data) [49].

**Volatile organic compounds (VOCs)**

To date, a total of 1,840 VOCs have been identified in various excretions of relatively healthy individuals on a common diet [50]. They include 872 compounds detected in exhaled air, 359 and 154 VOCs identified in the saliva and blood, respectively; 256 substances found in breast milk; 532 compounds obtained from skin secretions; and 279 and 381 VOCs detected in the urine and feces, respectively [50]. A CAS registry number (Chemical Abstracts Service registry number, a unique numerical identifier of a chemical substance) was assigned to each of these compounds.

The fecal metabolome is very rich in VOCs, which make up about 20% of the 1,890 unique compounds listed in the Human fecal metabolome database (HFMDB) [46]. Using various approaches to solid-phase microextraction of VOCs of the headspace above the feces of 17 healthy donors, Couch et al. revealed about 2,100 different compounds in total, many of which were identified based on data matches with the NIST database [51]. Acids and esters (> 550 metabolites), alcohols (> 450), alkenes (~ 400), alkanes (~ 300), aldehydes (> 250), and ketones (~ 200 metabolites) are the most common VOC classes in the feces of healthy donors. Various studies determined 80 to 300 VOCs on average by comparing the spectra and retention times with the data from known databases (e.g., GC–MS NIST, Wiley) [50-55].

A total of 297 [52] and 135 [56] different VOCs present in healthy donors were identified in two studies of the headspace above the feces. The results of these studies largely confirmed one another and at the same time revealed some differences. The average number of VOCs varied from 78 to 125 (median = 101). It is interesting to note that 44 of them were present in 80% of the donors and represented by ethanol, aldehydes and ketones (with a carbon chain length of 2–7), phenol, sulfur-containing compounds, and SCFAs [52].

It is extremely important to choose the proper method of fecal sample collection, since it has the potential to affect the results of the metagenomic and metabolomic analyses. Couch et al. [51] compared samples collected under different conditions (endoscopically collected and home-collected) and isolated using quick or long extraction protocols; the analysis revealed insignificant differences in their microbiomes and large differences in their metabolomes. A large portion of oxidized metabolites (alcohols, aldehydes, acids/esters) was observed in home-collected samples after a short (20- min) extraction, while reduced metabolites (alkanes, alkenes) predominated in the endoscopic samples. Since the VOC extraction profile is hyperbolic, prolonged (18- hour) extraction resulted in the identification of significantly more metabolites than short (20-min) extraction: 1,371/2,097 and 1,404/2,190 metabolites isolated by short/long extraction from endoscopically collected and home-collected fecal samples, respectively [51].

The origin of many fecal VOCs (whether they belong to the host organism or bacteria) and the metabolic pathways of their production have not been sufficiently studied. There is growing evidence that not only the individual nutritional status, but also gastroenterological disorders can change the VOC composition; therefore, VOCs may serve as potential diagnostic markers of gastrointestinal diseases [52-55].

**Short-chain fatty acids (SCFAs)**

SCFAs are organic fatty acids with a chain length of 1 to 6 carbon atoms; they are the main product of anaerobic bacterial fermentation of polysaccharides, proteins, peptides, and glycoproteins in the intestine. The main substrates for fatty acid synthesis are carbohydrates, mainly indigestible starches and dietary fiber, whose final fermentation product is mainly acetate, propionate, and butyrate [57]. In health, acetate, propionate and butyrate are present in the large intestine and feces in a constant molar ratio of 60: 20: 20 [33, 58]. Most of them are absorbed by host cells from the intestinal lumen [58].

Although the range of analytical methods for SCFA analysis has expanded significantly over the past decade, GC still remains the most frequently used method for quantitative determination of SCFAs in feces, despite a few disadvantages [33].

## METABOLITES AS POTENTIAL BIOMARKERS OF GASTROINTESTINAL TRACT DISEASES

The intestinal microbiome can be characterized by the composition of its metabolites. Clinical studies focuse on finding specific metabolites or unique metabolite combinations and metabolic profiles that can serve as disease biomarkers. Since feces constitute a complex and heterogeneous matrix, the data on them largely depend on both the interindividual variability and the capabilities of the analytical methods. Hence, a clear metabolic tendency may be hard to uncover when comparing data obtained through different methods on the same disease. Nevertheless, significant results have been accumulated to date, and they allow for considering fecal metabolite analysis as a new diagnostic tool.

The differential diagnosis of inflammatory bowel diseases (IBDs) remains complicated and usually relies on symptoms and examination results (laboratory tests, histological analysis, endoscopic and radiological examination) [59]. These diagnostic methods are often expensive and invasive. Serum markers, such as the C-reactive protein, are non-specific for IBDs and can also be detected in other inflammatory diseases [60]. The tests for determining the level of calprotectin and lactoferrin in a patient’s feces contribute to the IBD diagnosis [16, 61]. However, although these tests are informative, they are not specific because the proteins’ levels increase in other pathologies characterized by the presence of blood in the stool (hemorrhoids, polyps, or intestinal infections, e.g. *Clostridium difficile*) [62]. Thus, the tests cannot distinguish between infectious and non-infectious inflammatory diseases [62].

Several models that allow one to discriminate between patients with IBD from healthy donors [63-66] and IBS [66] using NMR spectroscopy have been proposed. In 2007, Marchesi et al. [63] were among the first to present an NMR-based characterization of fecal extracts of patients with CD and UC. Decreased levels of butyrate, acetate, methylamine, and trimethylamine, compared to the control group, were noted in this and subsequent studies, which correlated with changes in the gut microbial community and an increased content of amino acids (leucine, isoleucine, valine, lysine, alanine, tyrosine, phenylalanine, glycine, glutamate, and aspartic acid) due to the malabsorption caused by the inflammatory processes. Bjerrum et al. [64] attempted to differentiate between CD and UC using the metabolite analysis. However, removal of a significant group of patients with intestinal surgery and anti-TNF-α antibody therapy from the sample minimized the significance of the metabolic profiles of those patients. Thus, even minor intestinal surgery or drug therapy imposes significant individual imprints on metabolic profiles [64].

The main drawback of these studies is that metabolic models cannot differentiate between gastrointestinal diseases (e.g., Crohn’s disease and ulcerative colitis). Using a combination of various methods (NMR, LC–S, and GC–S) for the detection of non-volatile organic compounds does not help in solving the problem [65]. Santoru et al. [65] presented a comparative structural analysis of the metabolome of 183 stool samples (82 UC cases, 50 CD patients, and 51 healthy donors) by NMR, GC–S, and LC–S. Significant differences were found in the metabolic profiles of IBD patients and healthy donors. The NMR analysis turned out to provide the best prognostic score, as demonstrated by the Partial Least Square-Discriminant Analysis (PLS–A). The worst results corresponded to the LC–MS method. All three methods revealed comparable patterns of discriminatory metabolites in each of the diseases. The main metabolites were amino acids and their derivatives, fatty acids, trimethylamine oxide, B group vitamins (nicotinic and pantothenic acids). It is interesting to note that all three platforms failed to distinguish between the two pathological conditions (UC and CD), which indicates a significant intrinsic similarity between the metabolic profiles of these diseases [65].

The untargeted LC–MS analysis of 155 stool samples (68 CD cases, 53 patients with UC, and 34 healthy volunteers) revealed more than 8,000 low-molecularweight components, among which chemical classes and individual chemical compounds differentially present in IBDs were identified.

Metabolites (3,829, 43% of the total number) were ascribed to molecular classes based on matches with the HMDB 3.0 database and 346 unique compounds and then annotated as standards through a comparison with databases.

In general, the metabolic profiles of IBD patients (and especially CD patients) differed significantly from those of healthy volunteers. However, the localization of the inflammation did not affect the metabolic picture in CD. It should be noted that the UC patients’ metabolic profiles showed a broader distribution than those of CD patients, reflecting the profiles of both healthy volunteers and patients with CD. This may be associated with different levels of inflammation.

The level of primary bile (cholic and chenodeoxycholic) acids was significantly increased in CD, while the level of secondary (lithocholic and deoxycholic) acids was reduced. The levels of caprylic acid and fatty acids were decreased in the IBD group.

It is worth noting that patients with IBD often complain of an unpleasant fecal odor during disease exacerbation. Resident microflora is responsible for the fermentation of undigested food in the large intestine; it produces putrefactive compounds such as ammonia, aliphatic amines, branched chain fatty acids, indole, phenol, and volatile sulfur-containing substances, which affect both the intestinal state and metabolite composition. Therefore, disturbed intestinal microflora in IBD can result in altered stool odor [67, 68].

Apparently, accurate and reproducible detection of VOCs in biological samples has great potential in developing a non-invasive diagnostic test for IBD. To date, several studies comparing the VOC spectrum in feces, exhaled air, or the urine of IBD patients have been published [52, 53, 67, 69, 70]. Human feces are the final product of food intake, digestive and excretory processes, as well as bacterial metabolism [67]. Therefore, the analysis of fecal VOCs seems promising for gaining additional diagnostic knowledge.

Despite a limited sense of smell, medical personnel can diagnose a *C. difficile *infection by smelling a patient’s stool in 31 out of 37 cases [71]. Another study demonstrated that nurses could diagnose* C. difficile *with 55% sensitivity and 83% specificity [72]. However, it should be noted that trained dogs show significantly better results (83% sensitivity and 98% specificity) [73].

A GC–MS analysis of fecal VOCs allowed one to determine the differences between healthy donors and patients with IBS in [53], CD [54, 55, 70, 74], and UC [52, 55], discriminating between the patients with active and inactive CD [54, 55] and even between the patients with UC and an intestinal infection [29]. Garner et al. [52] compared the metabolic profiles of the patients with UC and *Campylobacter jejuni *and *C. difficile *infections and revealed the metabolites that distinguish infectious diseases from UC. For instance, 1-octen-3-ol is extremely common only in patients with *Camp. jejun*, although the origin of its overproduction has not yet been established. Similarly, the sulfur-containing compounds (dimethyl sulfide, dimethyl trisulfide, methanethiol) found in all samples obtained from the healthy donors were practically absent in the samples of patients with the *Camp. jejuni *and *C. difficile *infections [52].

In some cases, VOC profiling can even reveal the microbiological origin of the infection (viruses, bacteria, parasites). Robert et al. [75] found characteristic patterns of VOCs that depend on the etiology of infectious diarrhea. The presence of furan compounds is indicative of a *C. difficile *infection; ethyl dodecanoate is found in stool patents with rotavirus; and the absence of hydrocarbons and terpenes is a sign of *Campylobacter* infection [75].

In clinical practice, it is sometimes difficult to distinguish IBS patients who have the disease symptoms for the first time from IBD patients. VOCs of the superfluid gaseous fraction were analyzed, which made it possible to clearly distinguish IBS from IBD and healthy donors. Esters of short-chain fatty acids, cyclohexanecarboxylic acid, and its derivatives were present in excess and turned out to be the major discriminatory metabolites in IBS. The most common esters were the methyl esters of propionic and butyric acid [53].

The metabolite composition of the feces obtained from CD and UC patients in the active stage and remission, as well as healthy donors, was analyzed to locate discriminatory metabolites that are statistically significant in identifying the disease or its stage. The production of heptanal, propanal, benzeneacetaldehyde, 1-octen-3-ol, 3-methyl-1-butanol, 2-piperidinone, and 6-methyl-2-heptanone was significantly increased in the active CD group [54].

Aldehydes (heptanal, propanal, and benzeneacetaldehyde) are produced in inflammatory processes as a result of lipid oxidation and oxidative stress; they play an important role in tissue damage and ulceration of the gastrointestinal mucosa in IBD [76, 77]. Fecal aldehydes turned out to be more represented in active CD patients than in the relapse group and, especially, healthy donors. Therefore, they can serve as markers of disease activity. Secondary alcohols, 1-octen-3-ol and 3-methyl-1-butanol, were also found in maximum amounts in the acute CD patients. Moreover, 1-octen- 3-ol was not detected in the patients with active UC; therefore, it was regarded as a discriminatory VOC for the diagnosis of an active CD stage.

Aggio et al. used the electronic nose GC analysis and a computer algorithm for the study of fecal metabolites (33 active IBD patients, 50 inactive IBD cases, 28 IBS patients, and 41 healthy volunteers). The authors showed that it is possible to discriminate between active CD and IBS patients in 87% of cases and between IBS patients and healthy volunteers in 78% of cases [78].

Studies that seek to evaluate the effectiveness of the low-FODMAP (low fermentable carbohydrate) diet for patients with IBS can be widely used in practical medicine. A GC analysis, coupled with an electronic nose system, showed that it is possible to predict a favorable diet outcome based on a patient’s metabolic profile [79].

A quantitative determination of VOCs in biological samples is of great importance. Although the non-invasive biomarkers available to date can provide general information on the disease, they are not specific and cannot predict the disease course or possible complications. The diagnostic potential of using VOCs as noninvasive biomarkers for predicting risks, assessing the disease activity and therapy effectiveness is now under active study [80].

The electronic nose technology is being developed towards point-of-care portable sensor devices for real-time assessment of the state of the gastrointestinal tract and for diagnosing a disease by the uniqueness of the VOC profile [81, 82].

The accumulated experience allows us neither to use the metabolic profiles typical of specific diseases widely in clinical practice nor to discriminate between individual fecal metabolites for a diagnosis. Using wider panels of biomarkers, including both metabolites and macromolecules, which would reflect the multifactorial pathophysiology of the disease, seems an alternative. For instance, the use of individual non-invasive IBS biomarkers has yielded very moderate results so far. The developed panel of eight biomarkers (four plasma biomarkers: IL-1β, IL-6, IL-12, and TNF-α; four fecal biomarkers: chromogranin A, human β-defensin 2, calprotectin, and caproate) allowed researchers to diagnose IBS with a high level of confidence (88.1% sensitivity and 86.5% specificity) [83].

## CONCLUSION

Fecal metabolite analysis is a new branch of metabolomics which covers a wide range of compounds comprising readily-available biomaterial. Judging by the growing number of publications that appear year after year, it is obvious that fecal metabolomics has already assumed a position in this field of knowledge. The technological and instrumental progress achieved in the analysis methods used furthers this development. Although no reliable individual metabolic markers have Luzzatbeen identified to date, there are a number of general metabolic trends that can be clearly traced. They indicate lifestyle and diet features, the physiology of the intestine and intestinal microbiome, and the complex interactions between them in health and pathological conditions. The creation of the online Human Fecal Metabolome Database (HFMDB) became an important step in the use of the growing body of data, facilitating their interpretation. The database is continuously updated and already lists about 7,000 compounds, chemical and biological information on metabolites, as well as genes, proteins, metabolic pathways and possible associations with diseases.

Although the methods used to collect, process, and analyze fecal samples are developing rapidly, they remain unstandardized and do not always ensure a consistent interpretation of the results, which is a serious drawback. Feces have a heterogeneous composition and, thus, represent a complex study subject. This problem can be partially solved by using several analytical platforms in parallel or combining targeted and untargeted approaches, which significantly increase accuracy in the measuring of the metabolite level and reliability of the collected data. Developing unified approaches to the accurate quantitative assessment of metabolites using various analytical platforms would contribute to the further development of fecal metabolomics and its possible use in evidence-based medicine.

## Abbreviations

CD: Crohn’s disease
IBD: inflammatory bowel diseases
HPLC: high-performance liquid chromatography
GC–MS: gas chromatography coupled to mass spectrometry
GIT: gastrointestinal tract
LC–MS: liquid chromatography coupled to mass spectrometry
SCFA: short-chain fatty acid
VOC: volatile organic compound
IBS: irritable bowel syndrome
UC: ulcerative colitis
NMR: nuclear magnetic resonance
